# Exploring strategies for management of disasters associated with illegal gold mining in abandoned mines: A case study of Ekurhuleni Metropolitan Municipality

**DOI:** 10.4102/jamba.v14i1.1237

**Published:** 2022-03-11

**Authors:** Khodani Matshusa, Llewellyn Leonard

**Affiliations:** 1 Department of Environmental Sciences, College of Agriculture and Environmental Sciences, University of South Africa, Johannesburg, South Africa

**Keywords:** disaster management, abandoned mines, illegal gold mining, City of Ekurhuleni, South Africa

## Abstract

Due to many abandoned mines that are not rehabilitated, there will be illegal mining. Although the mining industry and government continue to prevent illegal gold mining in abandoned mines by sealing open shafts, it is not possible to close all the shafts at once due to limited resources. Furthermore, after sealing the shafts, illegal miners often create alternative openings to enter underground workings while little or nothing is being done to stop the surface illegal gold mining. As long as illegal gold mining is there, disasters associated with illegal mining are prevalent. Effective disaster preparedness and response requires a competent strategy. The purpose of this study was to develop a strategy that can be used for emergency preparation and rescue efforts associated with disasters caused by abandoned mines and illegal gold mining. In this qualitative study, semi-structured interviews were held with officials and experts on disaster management from the Council for Geoscience and the City of Ekurhuleni. This study indicates that the safety of illegal miners and communities near abandoned mines depends on several factors including the ability to identify and respond to a disaster. The study identified three interlinked themes within the report as strategies for dealing with disasters related to abandoned mines and illegal gold mining. These themes included emergency countermeasures and short-term measures, roles and responsibilities and communication. These interlinked themes should be validated through further research that involves input from the national disaster response community at large. This study will serve as a model that can be implemented in other areas impacted by illegal mining in South Africa.

## Introduction

South Africa has a long history of mining. Consequently, there are less than 2000 operating mines and close to 6000 abandoned mines in South Africa. South Africa is facing enormous financial, environmental and social challenges posed by abandoned mines (Matshusa & Makgae 2014; Cornelissen et al. 2019). ‘Because of socio-economic problems confronting communities around abandoned mine sites, historic and abandoned gold mines have become hot-spots for artisanal and small-scale miners’ (Mhlongo et al. 2019:1). Despite its importance as an economic activity and livelihood strategy in sub-Saharan Africa, illegal mining also referred to as artisanal and small-scale mining (ASM) is associated with many negative social, environmental and health impacts and presents particular sustainable development challenges (Collins & Lawson 2018; Kambani 2003; Lynas, Logrosa & Fawcett 2018). Abandoned mines are inherently dangerous to humans and the environment. For example, abandoned mines have resulted in soil pollution, air pollution through wind-blown dust from waste dumps, acid mine drainage (AMD) that contaminates drinking water for humans, unstable grounds and death of humans due to falling into dangerous mine openings and inhalation of dangerous gases (Matshusa & Makgae 2014; Liu et al. 2021; Salom & Kivinen 2020).

Although these negative challenges exist, illegal mining is on the rise and primacy in South Africa (Mhlongo et al. 2019; Phala, Mistry & Matlala 2017). While the individual illegal miners may act out of economic desperation due to poverty and job opportunities to sustain their livelihoods, illegal gold mining is directly linked to illicit trade of precious metals (Collins & Lawson 2018; International Criminal Police Organization (INTERPOL) 2021; Strydom 2016; Wagner 2016; Williams 2019). As long as abandoned mines are not rehabilitated, there will be disasters especially related to illegal gold mining. Disaster is a ‘situation resulting from an environmental phenomenon or armed conflict that produced stress, personal injury, physical damage, and economic disruption of great magnitude’ (Durga & Swetha 2015:516). Similarly, Mayner and Arbon (2015:24) define a disaster as ‘the widespread disruption and damage to a community that exceeds its ability to cope and overwhelms its resources.’ In relation to this study, a disaster is a social phenomenon caused by human activity (Al-Jazairi 2018). There is a lack of a clear academic definition of illegal mining in South Africa (Williams 2019) due to a lack of focused research on illegal mining. Furthermore, the distinction between illegal mining, artisanal mining or small-scale mining and informal mining is not well understood. According to Strydom (2016), the distinction between illegal and small-scale mining is that small-scale mining is regulated and encouraged while illegal mining is not. Phala et al. (2017) define illegal mining as conducting mining activities without a permit or license from the Department of Mineral Resources. Dozolme (2016) broadly proffered illegal mining as the absence of land rights, mining license, exploration or mineral transportation permit or any necessary documentation that could legitimise the mining. Williams (2019:12) contends that ‘if mining activities take place without the consent of the owners of the land or mining right, the activities can be considered to be illegal mining’. This study agrees that illegal mining is the act of mining without a mining license or permit while abandoned mine refers to a non-operating mine without owners or whose owners are not known or deceased or where the owners exist but the mine is temporarily not operational, or the owner cannot immediately carry out the rehabilitation of the site.

However, there is a lack of research focusing on disaster management especially for abandoned mines and illegal gold mining. The major outputs resulting from mine disaster prevention research are technical publications, presentations at professional meetings and proceedings (National Research Council and Institute of Medicine [NRCIM] 2007). Audiences are limited at technical symposia while ignoring the regulatory authorities of such municipalities. The previous research addresses some but not all of the high-priority areas; the strengths are in the area of mine fires and training for rescue teams (NRCIM 2007). There are no studies on the management of disasters from abandoned mines and illegal gold mining.

Following media reports highlighting the increased mine disasters related to illegal gold mining – especially across the Gauteng province, this study seeks to develop strategies to prevent and control mine disasters related to illegal gold mining conducted in abandoned mines in the City of Ekurhuleni (CoE). In South Africa government, mining companies, government agencies and researchers have focused on numerous aspects of mine emergency response but overlooked the strategies to prevent and control emergencies associated with abandoned mines and illegal gold mining. The South African Human Rights Commission (2015) and Mphokane (2018) identified that there is limited research dealing with the challenges of disasters related to illegal mining in South Africa. In this context, there is a need for further research that will consider strategies to address the issues and challenges related to abandoned mines and illegal gold mining (Matshusa & Makgae 2014). These include disasters such as crime, accidents and fatalities due to illegal mining within and around abandoned mines (Buxton 2013). Collectively, the estimates indicate that illegal mining or ASM operations worldwide experience six to seven times more accidents that occur at formal mining operations (International Labour Organisation 1999).

Moreover, no academic study has looked at strategies at both municipal and national levels to deal with disaster or emergency cases associated with abandoned mines and illegal mining. Some of the well-known incidents involving abandoned mines and illegal gold mining include the death of 76 illegal miners in 2009 from abandoned Harmony gold shaft (Reuters 2009), the death of a child by falling into an open mine shaft in 2017 at CoE (ENCA 2017) and the death of CoE metro police during a shoot-out with illegal miners in 2021 (Ngcobo 2021). These incidents have raised a number of new and re-occurring issues about abandoned mine emergency preparedness and response. In March 2017, approximately 1 month after the 2017 incident, the researchers conducted a study with experienced mine emergency responders of CoE and experienced scientists and management of the Council for Geoscience (CGS).

The goal of the research was to gather information to determine strategies for dealing with disasters related to abandoned mines and illegal gold mining in the CoE. This article, therefore, aims to explore the literature around abandoned and illegal gold mining and to propose strategies to combat disasters associated with abandoned mines and illegal gold mining. The article is structured into several sections including this introduction. The second section explores the literature surrounding abandoned mines and illegal gold mining by looking at the global cases in relation to South Africa, global challenges in mine disaster preparedness and regulatory framework in South Africa and communication in relation to disaster management. The third section describes the methodology employed. The results are discussed in the fourth section. Finally, the conclusions and recommendations are made in the fifth section.

## Literature review

### Abandoned mines and illegal mining: Global overview in relation to South Africa

Described by various terms (derelict, ownerless, abandoned, legacy, orphaned), abandoned mines are a global issue with millions of such sites likely to exist (Worrall et al. 2009). With various definitions, the common key characteristic of these sites is incomplete remediation of historical mines (Unger et al. 2015). ‘To protect the population and the environment in those cases, many countries are now undertaking clean-up projects financed by the government’ (Matshusa & Makgae 2014). Examples of these are the rehabilitation of Derelict and Ownerless (D&O) mines in South Africa (Matshusa & Makgae 2014; Cornelissen et al. 2019); United States of America (USA) Abandoned Mine Land Reclamation Program (AMLRP) and *Comprehensive Environmental Response, Compensation and Liability Act* (CERCLA); the National Orphaned/Abandoned Mines Initiative (NOAMI) in Canada (Tremblay & Hogan 2016); Australian Strategic Framework for Managing Abandoned Mines in the Minerals Industry (Unger et al. 2012); the ongoing programme for the German decommissioning of uranium mines and mills or Argentina’s Mining Environmental Restoration Program to remediate the closed uranium processing site in Malargüe, Mendoza (Fernandes et al. 2013). As long as abandoned mines are not rehabilitated, there will be illegal mining and associated disasters (Collins & Lawson 2018). However, this is not automatic as factors such as poverty, lack of job opportunities, illicit trade of precious metals (Collins & Lawson 2018; International Criminal Police Organization (INTERPOL) 2021; Strydom 2016; Wagner 2016; Williams 2019) may also lead to illegal mining.

In November 2020, the United Nations Office on Drugs and Crime (UNODC) indicated its support for combating illegal mining due to its negative environmental and economic impacts (UNODC 2020). According to International Criminal Police Organization (INTERPOL) (2021), illegal gold mining is associated with severe long-term environmental impacts, financial crimes, fraud, environmental crimes and trafficking in human beings. Furthermore, illegal gold mining can fuel insecurity and the presence of arms on mining sites posing serious security threats to law enforcement. The Global Initiative against Transnational Organised Crime carried out research on illegal mining in Latin America (Bolivia, Brazil, Colombia, Ecuador, Guyana, Mexico, Nicaragua, Peru and Venezuela) (Wagner 2016). According to Wagner (2016), illegal mining in Latin America is linked to organised crime. For example, in Peru, illegal mining is now more lucrative than drug trafficking although the two networks are becoming increasingly intertwined (UNODC 2020). In French Guyana, illegal mining has further wreaked havoc on the environment, through widespread pollution, erosion, water contamination and deforestation (UNODC 2020).

Illegal mining is widespread in Africa (SAHRC 2015) and increasing because of growing economic crises, unemployment and decreased rural livelihood (Collins & Lawson 2018). However, illegal miners have made ‘huge amounts of illicit profits at the expense of countries’ economies, vulnerable populations, and the environment across the Central African region’ (INTERPOL 2021:3). Illegal mining has plagued South Africa for decades, and according to the Chamber of Mines, South Africa (2016) has cost the government and the mining industry more than $1.3 billion USD a year in lost sales, taxes and royalties. According to the UNODC (2020), South African mining industry is worth $23.5 billion USD, but illegal surface and underground mining – and associated criminal activities have cost an estimated $8 billion USD in lost profit for the country annually despite best efforts to curtail the problem (UNODC 2020).

### Global challenges in mine disaster preparedness

Due to the increased number of mining disasters around the world, some countries have begun to recognise the need to have mine emergency rescue and disaster management plans (Dar et al. 2018). A critically important output was released to the industry by the National Institute for Occupational Safety and Health (NIOSH) in the USA to assess and immediately detect rock dusting (NRCIM 2007). The Coal Dust Explosibility Meter has the potential to prevent the propagation of explosions by enabling real-time evaluation of the explosibility of coal and inert dust mixes (Sapko & Verakis 2006). Other notable outputs include a computer-based emergency simulation exercise (Mine Emergency Response Interactive Training Simulation [MERITS]), the emergency communications triangle training materials focusing on the content of emergency warning messages and the evaluation of the lifeline and development of directional cones for self-rescue (NRCIM 2007).

The Quecreek mine inundation incident and the disasters at Sago and Alma Mine No. 1 revealed weaknesses in a number of disaster prevention and response areas (NRCIM 2007). Several of these can be addressed by a greater and more rigorous application of existing advances in disaster prevention and emergency response practices although research is needed on several fronts including disaster prevention, communications, escape and survival systems, and response equipment, rescue teams, *in situ* assessment of geologic conditions, monitoring of atmospheres behind seals and evaluation of mining methods (Ampofo & Adam 2018; NRCIM 2007). In all of these areas, there may be some potential for the transfer of technology and practices from the international community (e.g., Australia, Poland, South Africa, and Canada). The South African mining industry is one of the most developed in the world (Organisation for Economic Cooperation and Development 2002) and therefore has an important role to play in the management of disasters from abandoned mines. However, because of differences in laws, regulations and cultural or other practices, the direct application may not be feasible.

Communication has been recognised as a major source of problems in need of great attention to providing effective disaster response and escape (NRCIM 2007). The subject of emergency communications has been of particular concern for two committees of the National Research Council (NRC) that dealt with mine rescue and survival issues (National Academy of Engineering 1970; NRCIM 2007). In relation to abandoned mines, the *Pennsylvania Governor’s Commission on Abandoned Mine Voids and Mine Safety* (CAMVMS) addressed the issue of emergency communications (CAMVMS 2002). The 1981 NRC report pointed out a number of issues and potential approaches: underground-to-surface communications, communication between rescue teams and trapped miners and permanently installed seismic systems that could be used on a day-to-day basis. According to the CAMVMS (2002), there was no lack of basic technical knowledge, but the availability of practical engineering designs (which may have to be site-specific) was a major limitation. The NRCIM (2007) also emphasised the importance of the development of a communications system to each miner based on real-time data and analysis.

In the USA, where abandoned mines on government land are determined to need rehabilitation and reclamation, these are addressed, using a risk-based system, with funding provided by the coal-mining industry in terms of 1977 legislation (Bureau of Land Management n.d.). In Canada, the environmental legacies in respect of orphaned mines are the responsibility of the State. However, in some cases, partnerships with the private sector were addressed through an agreement between the government and relevant parties. In South Africa, the Department of Mineral Resources and Energy (DMRE) partnered with the CGS to rehabilitate and close all derelict and ownerless mine shafts. However, this is not enough due to limited financial resources (Matshusa & Makgae 2014). The recent mine disasters and the dramatic upsurge of disasters resulting in fatalities because of illegal gold mining in abandoned mines in South Africa must be reviewed.

### Regulatory framework in South Africa

Growing environmental awareness in South Africa during the 1990s (Matebesi 2020) resulted in new laws such as the *Mineral and Petroleum Resources Development Act* (MPRDA) of 2002; 2015 *National Environmental Management Act* (NEMA) of 1998 regulations on financial provision for prospecting, exploration and mining or production operations, being introduced for new mining projects. The legislations were aimed at amongst others to prevent and minimise the impacts of abandoned mines. However, because of the poor implementation of these laws, the impacts of abandoned mines are severe and exacerbated by activities of illegal mining (Mhlongo et al. 2019; Wagner 2016).

Illegal gold mining activities are taking place outside of the South African legal framework, particularly the MPRDA of 2002. According to the MPRDA, no person is allowed to extract or mine mineral and petroleum resources without a mining permit (Republic of South Africa 2002:s. 5). Illegal mining activities fall under schedule 1 of criminal offenses of the *Prevention of Organised Crimes Act* (POCA), 1998. In terms of POCA, illicit dealing in or possession of precious metals or precious stones is considered an offense (Republic of South Africa 1998). In terms of the *Prevention and Combating of Corrupt Activities Act* of 2004 (PCCAA), public officers are guilty if found engaged in corrupt activities (Republic of South Africa 2004). Arresting of crime syndicates fuelling illegal mining activities has proved useful but not enough is being done to arrest these syndicates (Chamber of Mines South Africa 2016). Therefore, this study proposes strategies for managing disasters in abandoned mines, especially those caused by illegal mining activities. However, illegal gold mining is not only affecting abandoned mines. Illegal miners are also operating were improperly sealed, abandoned tunnels meet operational tunnels of current gold mining companies (Harvey 2014). There are arguments that illegal mining should be formalised to leverage the economic benefits, save livelihoods and stimulate job creation from these activities (Azuma, Baah & Nachinaab 2021; Hosken & Bornman 2017; Masweneng 2017).

In case of a mine disaster, the DMRE has a legal obligation through the MPRDA to ensure that an investigation of the disaster is undertaken and reported on. However, there is uncertainty as to the responsible authority for reporting in the case of a disaster involving illegal gold mining. The DMRE and the South African Police Services (SAPS) are often found to contradict each other and are not sure who is responsible for reporting on the disaster. This can allow mining companies to evade their responsibilities with regard to environmental management and safety considerations of their mining operations (SAHRC 2015).

### Mine disaster and emergency planning in South Africa

Mine emergency plans are mandated in the South African mining industry at the national level (Republic of South Africa 2003:s. 38). However, there are no emergency plans for abandoned mines. All the mining companies are required to be trained to understand and follow the mine emergency plan (Bonsu et al. 2017; Mischo et al. 2014). Mining companies in South Africa are required to have a written emergency response plan that can be used immediately when a disaster occurs (Bonsu et al. 2017).

Mine disaster preparedness suggests a well-rehearsed, comprehensive emergency plan. A number of researchers support the notion that planning for an emergency is a process and follows a continual, dynamic cycle (Launhardt 2001; Pelfrey 2005; Perry & Lindell 2003). Perry and Lindell (2003) illustrate the relationships of three critical components of emergency preparedness: planning, the presence of written plans and training. Pelfrey (2005) suggests a model of preparedness that contains five broad phases including prevention, awareness, response, consequence management and recovery. Pelfrey (2005) contends the cycle allows for a dynamic, flexible and continuous process of interaction and integration, and functions as a self-organising mechanism that improves preparedness for anticipated events and for unimagined events. Launhardt (2001) suggests that all potential causes of a mine emergency must be identified before an emergency preparedness plan is developed. This study supports the concept of planning in advance and continually evaluating the emergency plan.

According to Orasanu and Connolly (1993), there are eight characteristics of real-world decision-making in response to a mine emergency: ill-structured problems; uncertain, dynamic environments; shifting, ill-defined, or competing goals; multiple event feedback loops; time constraints; high stakes; multiple actors; and goals of the organisation balanced against the decision maker’s personal choice. These characteristics support the concept of the advanced and continually planning process with respect to the dynamic environment, the presence of time constraints and the multiple event feedback loops demanding continual evaluation. Moreover, people tend to interpret events as normal as long as possible before defining the situation as out of the norm and needing action (McHugh 1968). As a first critical step in mine disaster and emergency response planning, thorough hazard analysis and risk assessment should be conducted (Launhardt 2001; Pelfrey 2005; Perry & Lindell 2003). Hopkins (2020) also describes this as a detailed investigation to understand what caused the disaster. According to Grayson (2001) risk in a mining context may be defined as a measure of both the likelihood and the consequences of a hazard associated with a mining activity or condition.

Roberts (2006) recommends developing a comprehensive emergency response plan that is risk based and mine specific. Grayson (2001) suggests that this can be achieved only if disasters are considered during planning and monitored through the mine operation. Valcik and Tracy (2017) found that while there are many different kinds of disasters, adequate planning and preparation for dealing with mine emergencies will ensure an effective response. An effective response, in turn, will allow the government and mine owners to protect and reduce the impact of the disaster on the local communities. Therefore, developing an effective disaster management and enforcement strategy can be an effective way of minimising disasters presented by illegal mining at abandoned mines (Environmental Protection Agency 2000).

### Communication

While communication has been recognised as one of the major challenges for disaster management, it is generally acknowledged that the most effective emergency communications system is one that is used for routine communication (Le Roux 2013; NRCIM 2007; Zamisa & Mutereko 2019). Therefore, the development of effective communications systems that would withstand the damaging effects of mine disasters is recommended (NRCIM 2007; Zamisa & Mutereko 2019). While no amount of good communication can make up for poor emergency planning, communication can prevent misunderstandings and build credibility (EPA 2000). In this context, effective communication is essential for the safe function of mine rescue and disaster management team (Enright & Ferriter 2015; NRCIM 2007) and trapped or involved illegal miners or communities at an incident like a mine rescue from abandoned mines.

Mine disasters that require emergency rescue teams to conduct search and rescue operations require special communication equipment such as mine phones, leaky feeder systems, medium frequency radios and through-the-earth (TTE) communications (Enright & Ferriter 2015). According to the EPA (2000:5-4), ‘it is also important to identify and communicate through the community’s informal networks using unofficial community caretakers and opinion-makers.’ It is important to develop an integrated communication approach to bridge the communication gap and ensure knowledge sharing between local communities and other organisations such as national government departments and municipalities (Brockmyer & Fox 2015; Lynas et al. 2018). This will ensure trust amongst all stakeholders and ensure that disasters are reported early to fast track and improve the chances of emergency rescue efforts to be successful.

## Research methods and design

This study comprised a comprehensive review of the abandoned mines and illegal gold mining literature, perceptions of participants through semi-structured interviews. It drew heavily on themes identified from the analysis of semi-structured interviews with key informants in government (Table 1). This study specifically focuses on the analysis of interviews with key informants in government, thus, CGS and CoE, and their reflections on new emergency measures to improve disaster prevention, rescue and recovery efforts associated with abandoned mines and illegal gold mining.

**TABLE 1:** Characteristics of key participants (*n* = 10) in government who participated in the semi-structured interviews.

Participants involved	Role	Highest level of education	Age group	Gender	Race
CGS official (DS)	Head of Unit	PhD	50–59	Male	Black
CGS official (VVRK)	Project Manager	PhD	50–59	Male	Indian
CGS official (CSJ)	Senior Scientist	Masters	30–39	Male	Black
CGS official (MB)	Team Leader	Honours	20–30	Female	Black
CGS official (MD)	Project Manager	Degree	30–39	Male	Black
CGS official (NNJ)	Junior Scientist	Honours	20–29	Male	Black
CoE official (NWM)	Communication	Not provided	40–49	Female	Black
CoE official (TS)	Manager: Disaster Management	Not provided	40–49	Male	Black
CoE official (MA)	Disaster Management Official	Not provided	40–49	Male	Black
CoE official (MP)	Project Manager	Masters	30–39	Male	Black

Note: DS, VVRK, CSJ, MB, MD, NNJ, NWM, TS, MA, and MP are codes used for participants to hide their identities.

CoE, City of Ekurhuleni; PhD, Doctor of Philosophy.

A purposive sampling strategy was used to gather the data. Participants were selected based on their unique knowledge and understanding of the topic (Gentles et al. 2015; Marshall 1996) and to ‘achieve representativeness’ (Maxwell 2009:235). The sample size was determined by the willingness and availability of the participants to take part in this study. The semi-structured interviews were open ended in nature to elicit views and opinions from the participants. Using open-ended questions facilitated interactive discussion, and the participants were able to freely express their opinions (Albudaiwi 2017; Roulston 2008). Predetermined key questions asked during the semi-structured interviews were based on the constructs of abandoned mines and illegal gold mining, which informed the interview guide (Edwards & Holland 2013). Interviews were stopped when researchers reached saturation of data from the key informants. The semi-structured interviews sought to elicit information on challenges related to, abandoned mines and illegal gold mining; emergency and disaster management and communication. This study examines the themes that emerged from interviews with 10 key participants in government. The CGS was represented by six participants and CoE was represented by four participants (Table 1).

The data was analysed using a manual content analysis approach whereby the data were analysed according to categories and topics that emerged from the data rather than making *a priori* assumptions. Creswell’s six steps of data analysis and interpretation were followed (Creswell 2014), namely (1) organise and prepare the data for analysis, (2) read or look at all the data, (3) start coding all the data, (4) use the coding process to generate a description of the setting or people as well as categories or themes for analysis, (5) advance how the description and themes will be represented in the qualitative narrative and (6) interpret the data. The identified themes were used to determine the strategy for disaster management related to abandoned mines and illegal gold mining in the CoE.

### Ethical considerations

All procedures performed in studies involving human participants were in accordance with the ethical standards of the institutional and/or national research committee and with the 1964 Helsinki Declaration and its later amendments or comparable ethical standards. Informed consent: Verbal informed consent was obtained from all individual participants involved in the study. This was because the participants did want to be quoted verbatim and sign the consent form.

## Results and discussion

Three broad interlinked themes listed below emerged from the data analysis process:

- emergency countermeasures and short-term measure
- roles and responsibilities
- communication.

### Emergency countermeasures and short-term measures

The emergency countermeasures and short-term measures theme emerged as the semi-structured interviews discussed the strategies to deal with the challenges of disasters related to abandoned mines and illegal gold mining. All the participants had the same understanding that illegal mining refers to mining without a mining permit. All the participants recognised that proactive disaster preparedness is critical for the effective implementation of disaster prevention and mitigation strategies. The results indicate the lack of documented emergency countermeasures at both municipal and national levels to deal with emergency cases related to illegal gold mining at abandoned mines. The participants agreed that as a matter of urgency, the following emergency countermeasures are required:

- Communication and public awareness about the dangers associated with abandoned mines and illegal gold mining, especially those close to communities and easily accessible.
- Establishment of a multi-skilled emergency response team to coordinate activities that include people from CGS, CoE and DMRE.
- Creation of an emergency fund (this funds should be ring fenced and readily available) to effectively implement the proposed emergency countermeasures and short-term measures.
- Appointment of preferred emergency suppliers to avoid procurement delays with implementing emergency countermeasures and short-term measures.
- Provision of resources such as equipment and manpower by the affected municipality.
- Approval of deviation from the normal procurement process.

The participants did agree that short-term emergency measures during disasters management associated with abandoned mines and illegal gold mining should also be considered. The short-term measures suggested include:

- palisade fencing with signage
- sealing of the mine shafts or openings with appropriate temporary structures
- razer barbed wire and
- public awareness.

This study acknowledges that other participants such as DMRE may have different views from the CGS and CoE. As such, Matshusa (2017) show the viewpoints of DMRE. According to Matshusa (2017), DMRE is like a government mafia and dominates other local and provincial governments. This may be because of political interference and corruption by government officials in soliciting economic gains from illegal mining activities. As Matshusa (2017) highlighted that corruption in government is hindering effective governance in mining development.

After considerations of factors such as theft and availability of financial resources, it was recommended that an adequate combination of some or all four short-term measures above be implemented. This is in line with previous research that showed that the combination of demarcating and rehabilitating abandoned mines, and public awareness would provide a balance between emergency countermeasures and short-term measures (Collins & Lawson 2018; Phala et al. 2017).

The officials from CoE noted that the lack of capacity to deal with emergencies such as cases of disasters related to abandoned mines and illegal gold mining, and therefore it is critical that it partners with other government institutions and SoEs that have expertise and resources to manage these challenges. This study corroborated previous research regarding the need for funds and officials to undertake capacity-building courses related to emergency countermeasures and short-term measures for disasters associated with abandoned mines and illegal mining (Collins & Lawson 2018; Mphokane 2018; Valcik & Tracy 2017). Furthermore, there was a lack of capacity in human resources at all spheres of government that resulted in ineffective governance (Matshusa 2017) to effectively manage disasters related to illegal mining.

### Roles and responsibilities

The roles and responsibilities theme was identified when discussing the responsible institutions when responding to disasters related to abandoned mines and illegal gold mining. Perceptions on institutional preparedness and legal standing to take responsibility were considered. In this case, the roles of the national government, municipalities and government entities should be clearly defined by an ‘emergency plan of action, which should allow for cooperation and resources to be utilized effectively’ (Valcik & Tracy 2017:7). In this study, all participants agreed that in the case of a disaster associated with abandoned mines and illegal gold mining, the overall responsibilities lie with DMRE as the custodian of mineral resources according to the MPRDA. All participants agreed that the CGS will receive instructions from DMRE to conduct any task relating to such emergency in accordance with the *Geoscience Act*, No. 100 of 1993 as amended. The CoE remains the face of the government and should remain responsible for communication with the public.

All participants agreed that the executives of the three institutions (DMRE, CGS and CoE) should determine the sources and methods of funding. This will also ensure the coordination of disaster or emergency management activities within the government entities and other stakeholders (Phala et al. 2017; SAHRC 2015; Valcik & Tracy 2017). According to Valcik and Tracy (2017):

[*I*]neffectual coordination can result in overlapping responsibilities among different organisations, which can lead to bickering and wasted efforts, or ineffective control, which can lead to gaps in capabilities and responses. (p. 15)

Thus, there is an urgent need to rebalance roles and responsibilities amongst government departments to enable co-operative and effective governance (Matshusa 2017).

### Communication

The communication theme emerged as participants discussed the surrounding challenges on information sharing, awareness as well as techniques to convey information about disasters associated with abandoned mines and illegal gold mining. Participants from the CGS highlighted that there is no formal platform for sharing critical information and knowledge on disasters related to abandoned mines and illegal gold mining at CoE. This supports research by Hopkins (2020:23) that ‘one of the recurrent findings in disaster research is that information that something was wrong was available somewhere within the organisation but was not communicated to relevant decision-makers’. Furthermore, there is a need to raise awareness about the dangers of illegal mining (SAHRC 2015).

As such, all participants recognised that effective communication is a key to successful disaster management. Government participants highlighted that they do community engagement (Matshusa 2017) on issues of mining development including illegal mining in abandoned mines. However, participants from CGS and CoE highlighted that the engagement by DMRE is limited as the views of local and provincial government departments are not taken into account. This supports the study by Matshusa (2017) that collective input from all concerned government departments was not possible because of lack of communication, cooperation by DMRE during decision-making and participatory process. Thus, participants agreed that, when an emergency situation occurs, there should be a communication framework in place to ensure that appropriate decisions are taken and implemented on time. As a result, communication should also involve public awareness about the dangers associated with abandoned mines and illegal gold mining, especially those that are close to communities and easily accessible. Communication will improve the relationship between local communities, local authorities and the government (Collins & Lawson 2018; Mphokane 2018; Phala et al. 2017).

Furthermore, participants highlighted the need to create emergency email and SMS groups. The email and SMS groups should include responsible people from relevant organisations, and in case of a disaster, CoE should communicate with the group via email as soon as an incident is reported. This communication system will ensure that important information is highly visible and that all relevant decision-makers will become aware of it (Hopkins 2020). Furthermore, the communication system would through engagement with both government officials and community members, through multi-stakeholder workshops and reflection groups promote community development and a sense of social responsibility amongst entities (Collins & Lawson 2018; Mphokane 2018; Phala et al. 2017).

However, participants from the CGS indicated that they will only be involved in disasters and emergency cases that are aligned with its mandate according to the *Geoscience Act*, No. 100 of 1993. It is recommended that an emergency should be communicated within 12 h after its occurrence to allow proper implementation of short-term measures.

## Conclusion

This study indicates that the safety of illegal miners and local communities near abandoned mines depends on several factors including the ability to identify and respond to a disaster. Participants were able to identify three interlinked themes (emergency countermeasures and short-term measures, roles and responsibilities and communication) as strategies for dealing with disasters related to abandoned mines and illegal gold mining. The theme on emergency countermeasures and short-term measures must ensure proactive disaster preparedness, prevention and effective rescue. Roles and responsibilities theme in decision-making in relation to the control of disasters associated with abandoned mines and illegal gold mining must be structured so that the ‘do nothing’ option is removed. This involves the development of plans for the management of abandoned mines and illegal gold mining disasters with information on who is responsible for carrying out these responsibilities. The theme on communication ensures that there are processes to guarantee effective information sharing, awareness as well as techniques to convey information about disasters associated with abandoned mines and illegal gold mining between all decision-makers and other stakeholders. To enable efficient sharing of information and resources, it is recommended that means of co-operation such as a Memorandum of Understanding (MoU) or any other means of co-operation should be developed and signed between CoE, CGS, DMRE and any other involved stakeholders. This will serve as the basis for future engagements and cooperation with other affected municipalities in South Africa. These interlinked themes should be validated through further research that involves input from the national disaster response community at large.
